# Housing Demand in Urban Areas and Sanitary Requirements of Dwellings in Italy

**DOI:** 10.1155/2020/7642658

**Published:** 2020-02-27

**Authors:** Marco Dettori, Lucia Altea, Donatella Fracasso, Federica Trogu, Antonio Azara, Andrea Piana, Antonella Arghittu, Laura Saderi, Giovanni Sotgiu, Paolo Castiglia

**Affiliations:** ^1^Department of Medical, Surgical and Experimental Sciences, University of Sassari, Sassari 07100, Italy; ^2^Department of Architecture Design and Urban Planning, University of Sassari, Sassari 07100, Italy; ^3^Department of Prevention—Hygiene and Public Health Service, Sassari 07100, Italy; ^4^Department of Biomedical Sciences, University of Sassari, Sassari 07100, Italy

## Abstract

The phenomenon of urbanisation is becoming increasingly prevalent on a global level, and the health issues regarding the urban environment are of primary importance in public health. Accordingly, the present manuscript describes an analysis of the housing conditions of Italian urban areas, referring to the city of Sassari (Sardinia), Italy, focused on the dwelling structural and sanitary conditions issued by the Italian regulations. Data relating to the housing conditions of the population were acquired by the Local Hygiene and Public Health Service (SISP), in a period between 2012 and 2016. Qualitative variables were summarised with absolute and relative (percentages) frequencies, whereas quantitative variables with means and standard deviations depending on their parametric distribution. Statistical comparisons for qualitative and quantitative variables were performed with the *χ*^2^ test or Student's *t*-test, respectively. A *p* value less than 0.05 was considered statistically significant. Finally, the dwellings and the collected variables were georeferenced on a city map. During the 2012–2016 observation period, 363 certification requests were received from 193 (53.2%) foreign-born citizens and 170 (46.8%) Italians at the SISP offices. The main reasons relate to the request for a residency permit (46.6%) and to obtain a subsidy from the local government (32.8%). Overall, 15.4% of dwellings were found to be improper, while 35.3% and 22.0% were found to be unhygienic and uninhabitable, respectively. The foreigners' homes were found to be suitable in 82.7% of cases; the housing of Italian citizens, on the contrary, was found to be suitable in 28% of the observations. The present study offers a cross section of the housing conditions of Italian urban areas, referring to the city of Sassari. To the authors' best knowledge, this observation is the first one carried out in Sardinia and one of the first observations in Italy. It has emerged that “hygienically unsuitable” homes are those that, in most cases, are located in the city centre. Moreover, the Italian population is hit by a significant housing problem, due to overcrowding, uninhabitability, and unhygienic conditions. Overall, our findings suggest that it is necessary to develop a multidisciplinary approach to guarantee public health, with safe dwellings homes and the surrounding urban context alongside the development of social relations. Nevertheless, there is still little evidence available today on the population housing conditions, especially regarding the private indoor environment, and further research is needed to bridge this knowledge gap.

## 1. Introduction

Urbanisation is increasing worldwide, with 70% of the global population estimated to live in urban areas in the near future [1, 2].

Health issues in the urban environment are partially addressed [3]. Observational studies on the sanitary conditions of civilian homes are still lacking. Housing can be a key determinant of health [4]: it was estimated that unsuitable housing conditions (e.g., overcrowding) can cause unhealthy conditions [5], costing the European Union ∼194 billion euros annually [6].

A recent survey [7] showed that 16.6% of the European population was living in overcrowded conditions in 2017. In Italy, 27.8% and 7% of the population lived in overcrowded or overcrowded and damp (e.g., no bathrooms and poor lighting), respectively [7]. It was proved the association of those conditions with low income and social phenomena (e.g., divorces and separations) linked to unstable individual economy [8–11].

Poverty can be indirectly proved by the difficulty of maintaining or accessing suitable accommodation, with a consequent increase in public or private housing demand.

However, following the poor availability of social housing, people often resort to renting private accommodation without a legally valid contract, while the phenomenon of squatting increases living in unhealthy and unsafe places [12, 13].

As a further complication, the Italian regulatory system on minimum health and hygiene requirements of civilian homes is quite inconsistent. Although an *ad hoc* legislation has been in force since 1975 [14], other national and regional regulations affect its systematic implementation [15].

To address the growing housing demand, several sanctions and amnesties (i.e., Law 47/85) [16] have been adopted [17, 18].

The Sardinian region issued the Regional Law no. 21 in 2011 [19] for the use of semibasements and attics as dwellings. However, the occurrence of the cyclone Cleopatra in 2013 killed families living in semibasements [20].

The aim of the study was to assess housing conditions of the population residing in urban areas of Sardinia, Italy, between 2012 and 2016, in order to evaluate (i) dwelling structural and sanitary conditions based on the requirements issued by the Italian regulation; (ii) if differences exist between dwellings conditions; and (iii) the most important variables associated with those differences.

## 2. Materials and Methods

Data were collected from the urban area of Sassari, Italy, from 2012 to 2016. Sassari is a city located in the northeast of the region and is the second largest city in Sardinia, both for its surface area (546.08 km^2^) and number of inhabitants. Although the Municipality of Sassari has a considerable size, the urban nucleus contains 78% of the resident population (>100,000 inhabitants) [21]. According to data reported by the Italian National Institute of Statistics (ISTAT), the mean (standard deviation, SD) resident population during the observation period was 126,603 (1,316) inhabitants [21]. The mean (SD) foreign population residing between 2012 and 2016 was 3,375 (578). An increasing trend was described in both native and migrant populations during the study period. The majority of foreign citizens reside in the city's historical centre, characterised by a medium-high index of social deprivation [22].

Information on housing conditions was acquired by the Local Hygiene and Public Health Service (SISP) (i.e., housing suitability requests). An investigation was carried out by the SISP health personnel prompted by the request of dwelling's suitability certification, voluntarily submitted by the inhabitants: it assessed dwellings' structural and sanitary conditions prescribed by the Italian regulation on living environments using an ad hoc form [14]. Collection of comprehensive information on living conditions of families and individuals residing in the Municipality was not achievable: only data from those needing certification were retrieved.

The study did not require ethical approval for its observational design according to the Italian law (Gazzetta Ufficiale no. 76 dated 31.3.2008).

SISP health personnel collected information on the country of origin, the reason behind the request, the dwelling location, and the requirements shown in Table 1.

According to the findings of the investigation, the health personnel issued a final decision on the dwelling. In particular, a dwelling was defined: improper if deemed unsuitable and did not show the adequate characteristics for an accommodation; uninhabitable if the dwelling conditions were unhealthy; suitable if all legislative requirements were addressed.

Data were entered on Excel (Microsoft Office, Microsoft Corporation, Redmond, WA, USA) and analysed using the STATA software 15 (StatCorp., Austin, TX, USA). Qualitative variables were summarised with absolute and relative (percentages) frequencies, whereas quantitative variables with mean and standard deviation were summarised depending on their parametric distribution. Statistical comparison for qualitative and quantitative variables was performed with the *χ*^2^ test or Student's *t*-test, respectively. A *p* value less than 0.05 was considered statistically significant.

The ArchGIS software (https://www.esri.com/en-us/home) was used to georeference dwellings and the collected variables on a city map.

## 3. Results and Discussion

During the 2012–2016 period, 363 certification requests were received from 193 (53.2%) foreign citizens and 170 (46.8%) Italians (Table 2).

Requests ranged from 136 in 2012 to 37 in 2016, with a minimum of 18 requests in 2015. The main reasons relate to the request for a residency permit (46.6%) and to obtain a subsidy from the local government (32.8%). In total, 56 (15.4%) dwellings were found to be inadequate, 128 (35.3%) unhygienic, and 80 (22.0%) uninhabitable. Furthermore, about 9% of homes were overcrowded, 18.2% were poorly illuminated, and 30.6% were found to have traces of damp.

Differences between housing conditions of dwellings of Italian citizens (worse) and those of foreigners were significant (Table 3).

**Figure 1:**
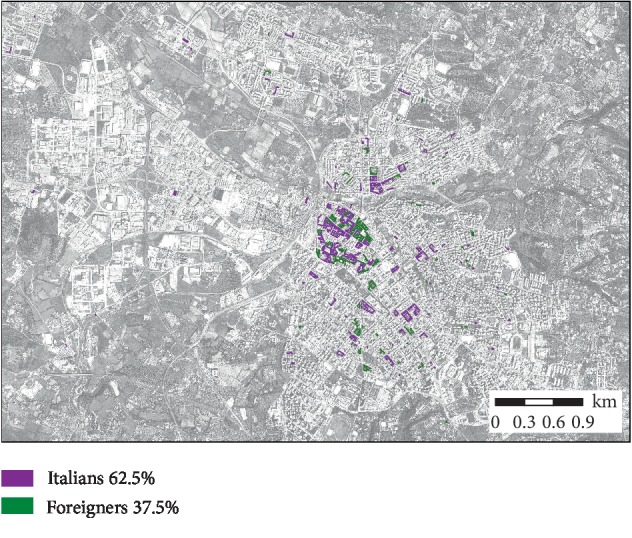
Dwellings of Italian and foreign citizens requesting housing suitability certification.

Dwellings subject to certification were often located in the historical centre of the city (Figure 1).

**Figure 2:**
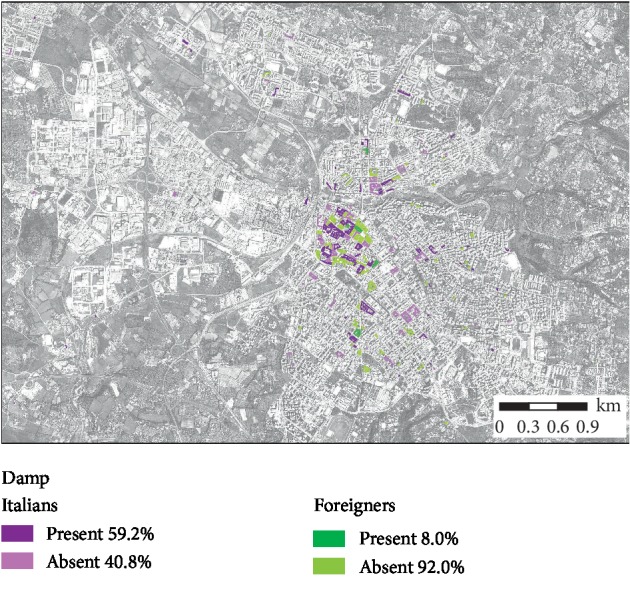
Damp presence per dwellings for Italian and foreign-born citizens.

**Figure 3:**
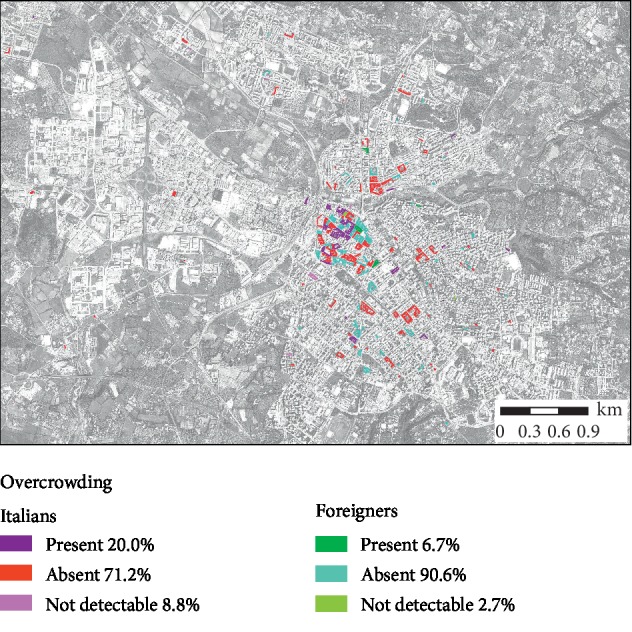
Overcrowding per dwelling for Italian and foreign-born citizens.

Figures 2 and 3 highlight the characteristics of homes in relation to overcrowding and the presence of damp, for Italian and foreign-born inhabitants, respectively.

A total of 56 turned out to be inadequate housing; 18 (14.4%) inhabited by Italians and 5 (6.7%) inhabited by foreign-born individuals were unhealthy (attics, basements, and warehouses), characterised by the absence of a bathroom, kitchen, and running water.

**Figure 4:**
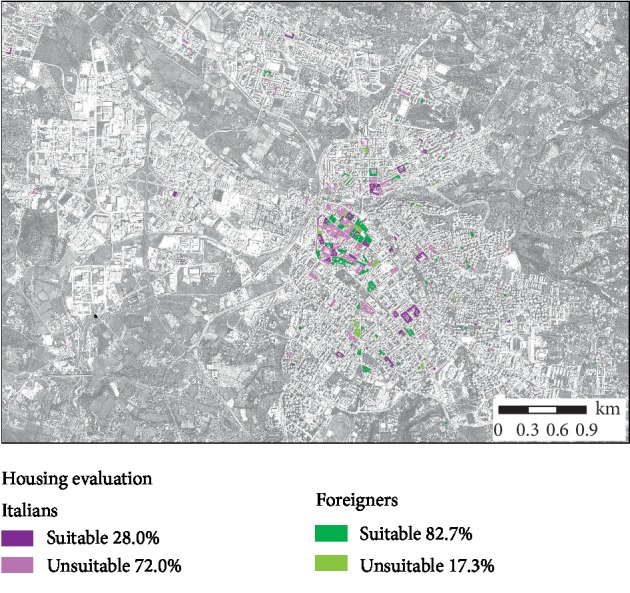
Sanitary evaluation of dwellings inhabited by Italian and foreign-born citizens.

Foreigners' and Italians' houses were found to be suitable in 82.7% and 28% of the cases, respectively (Figure 4).

## 4. Conclusions

The present study shows housing conditions of an Italian urban area.

40.8% of houses were hygienically unsuitable. This result mainly depends on the characteristics of the houses of the autochthonous population. Our data, in line with those of other national studies [23], are probably attributable to the anxiety of the obtaining subsidies from local government for precarious housing conditions for the native population; on the other hand, foreigners needing a residence permit should prove that they own an adequate accommodation. On this basis, 82.7% of the dwellings inhabited by foreign-born citizens were found to be suitable, whereas only 28% of dwellings inhabited by the native population were found to be suitable.

Furthermore, overcrowding, uninhabitability, and unhygienic conditions were described, as well as damp dwellings and poor window/floor ratios.

Hygienically unsuitable houses are located in the city centre, where buildings have significant structural and hygienic deficiencies.

This finding is striking if compared with traditional conception of the European city centre where buildings are in better conditions, with public spaces, quality services, and prestigious institutional locations [24, 25].

Historic city centres usually constitute an architectural heritage. However, the historical centre of Sassari is a largely deprived area, affected by urban discomfort and social exclusion [26], with shortcomings related to poor structure and dated buildings.

Therefore, it is key to plan adequately, regenerating historical areas and creating and recovering environmental spaces which could fit structural, hygienic, and health requirements [27, 28].

The Italian Ministry of Health Decree of 5 July 1975 [14], which states the health and hygiene requirements for civilian houses, is often circumvented by other legislative measures (e.g., Law 47/85 on building sanction and amnesty) [16]. Furthermore, DPR 425/94 [29] stated that hygienic-sanitary assessment on housing suitability can be replaced by a self-certification of conformity to the standards by the designers.

Awareness and education on health risks related to the unhealthy, unsafe, and unsanitary housing conditions are crucial for a rapid change of the current situation [29]. Indeed, studies showed that public perception of potential domestic risks is altered in relation to the actual incidence of events [30–37].

A multidisciplinary is required (architects, urban planners, sociologists, hygienists, psychologists, local authorities, etc.) to address the main public health issues related to environmental risks, including those associated with safety and hygiene of domestic houses [38–43].

Several limitations can be found: only dwellings voluntarily subjected to verification were recruited in the study design (potential selection bias). However, those results can be helpful for policymakers and politicians, as well as public health specialists, for framing and planning new activities under the principles of “health everywhere and for everyone.” In particular, data can be used to create indicators for cost analysis, assessment of the efficiency of an intervention, and assessment of models of social and economic costs. There is still poor evidence on the population housing conditions, especially regarding the private indoor environment. The study period was too short and cannot describe temporal variability for the purpose of comparisons. Furthermore, the Italian decree was issued in 1975 and, then, the inclusion of more appropriate variables is needed. Further research is needed to address the current knowledge gaps and to improve the abovementioned limitations (e.g., inclusion of other descriptive variables, inclusion of voluntary and non-voluntary reports, and increase in the duration of the study period).

## Figures and Tables

**Table 1:** Dwelling requirements evaluated.

Requirement issued by Health Ministry Decree [14]	Description
*Poor ventilation and illumination*	Window/floor surface ratio lower than 1 : 8
*Damp*	Presence of damp on vaults and walls
*Incomplete/absent services*	Absence or incomplete presence of bathrooms, kitchens, running water
*Unhygienic dwelling*	Presence of signs of damp not treatable with simple surface maintenance, scarce window/floor surface ratio, presence and quality of the heating systems, and room floor-size
*Overcrowded dwellings*	No. of individuals residing in the dwelling in relation to its surface area higher than the value envisaged by articles 2-3 of the 1975 Ministerial Decree

**Table 2:** Descriptive analysis.

Variables		Frequency (%)
*Year of certification request, no. (%)*	*2012*	136/363 (37.4)
	*2013*	135/363 (37.2)
	*2014*	37/363 (10.2)
	*2015*	18/363 (5.0)
	*2016*	37/363 (10.2)
*Italians, no. (%)*		170/363 (46.8)
*Reason for the request, no. (%)*	*Residency permit*	169/363 (46.6)
	*Subsidy*	119/363 (32.8)
	*Provincial labour Office request*	48/363 (13.2)
	*Social housing requirements*	21/363 (5.8)
	*Family reunification*	3/363 (0.8)
	*Adoption*	2/363 (0.5)
	*Hospitality*	1/363 (0.3)
*Improper dwellings, no. (%)*		56/363 (15.4)
*Poor ventilation and illumination, no. (%)*		66/363 (18.2)
*Damp, no. (%)*		111/363 (30.6)
*Incomplete/absent services (bathrooms, kitchens, running water), no. (%)*		40/363 (11.0)
*Unhygienic dwelling, no. (%)*		128/363 (35.3)
*Overcrowded dwellings, no. (%)*		32/363 (8.8)
*Uninhabitable dwellings, no. (%)*		80/363 (22.0)
*Suitable dwellings, no. (%)*		215/363 (59.2)

**Table 3:** Analysis of the differences between the housing conditions of the dwellings of Italians and foreign citizens.

Variables			Foreigners (no. 193)	Italians (no. 170)	*p* value
*Year of certification request, no. (%)*		*2012*	80/193 (41.5)	56/170 (32.9)	0.002
		*2013*	79/193 (40.9)	56/170 (32.9)	
		*2014*	18/193 (9.3)	19/170 (11.2)	
		*2015*	4/193 (2.1)	14/170 (8.2)	
		*2016*	12/193 (6.2)	25/170 (14.8)	
*Reason for the request, no. (%)*		*Residency permit*	163/193 (84.4)	—	**<0.001**
		*Subsidy*	17/193 (8.8)	105/170 (61.7)	
		*Provincial labour office request*	8/193 (4.2)	41/170 (24.1)	
		*Social housing requirements*	2/193 (1.0)	20/170 (11.8)	
		*Family reunification*	3/193 (1.6)	—	
		*Adoption*	—	3/170 (1.8)	
		*Hospitality*	—	1/170 (0.6)	
*Improper dwellings, no. (%)*			10/193 (5.2)	46/170 (27.1)	**<0.001**
*Poor ventilation and illumination, no. (%)*			11/193 (5.7)	55/170 (32.4)	**<0.001**
*Damp, no. (%)*			17/193 (8.8)	94/170 (55.3)	**<0.001**
*Incomplete/absent services (bathrooms, kitchens, running water), no. (%)*			7/193 (3.6)	33/170 (19.4)	**<0.001**
*Unhygienic dwelling, no. (%)*			20/193 (10.4)	108/170 (63.5)	**<0.001**
*Overcrowded dwellings, no. (%)*			10/193 (5.2)	22/170 (12.9)	**0.009**
*Uninhabitable dwellings, no. (%)*			16/193 (8.3)	64/170 (37.7)	**<0.001**
*Suitable dwellings, no. (%)*			167/193 (86.5)	48/170 (28.2)	**<0.001**

## Data Availability

Datasets used and/or analysed during the current study are available from the corresponding author upon reasonable request.

## References

[1] World Health Organization (2019). *Urban Health*.

[2] United Nations General Assembly (2015). *Transforming Our World: The 2030 Agenda for Sustainable Development*.

[3] World Health Organization (2010). *Why Urban Health Matters? World Health Day 2010*.

[4] Dahlgren G., Whitehead M. (2007). *European Strategies for Tackling Social Inequities in Health: Leveling Up, Part 2*.

[5] World Health Organization (2018). *Housing and Health Guidelines*.

[6] Eurofound (2016). Inadequate housing in Europe: costs and consequences. https://www.eurofound.europa.eu/publications/report/2016/quality-of-life-social-policies/inadequate-housing-in-europe-costs-and-consequences.

[7] Eurostat (2018). *Housing Statistics*.

[8] Braubach M., Fairburn J. (2010). Social inequities in environmental risks associated with housing and residential location--a review of evidence. *The European Journal of Public Health*.

[9] ISTAT (2018). Bilancio demografico nazionale. Anno 2017.

[10] ISTAT (2016). Matrimoni, separazioni e divorzi. https://www.istat.it/it/files//2016/11/matrimoni-separazioni-divorzi-2015.pdf.

[11] D’Alessandro D., Raffo M. (2011). Adapting the answers to new problems of living in a changing society. *Annali Di Igiene*.

[12] Federcasa (2016). Dimensione e caratteristiche del disagio abitativo in Italia e ruolo delle aziende per la casa. https://www.federcasa.it/wp-content/uploads/2017/03/Disagio_abitativo.pdf.

[13] Capasso L., Gaeta M., Appolloni L., D’Alessandro D. (2017). Health inequalities and inadequate housing: the case of exceptions to hygienic requirements for dwellings in Italy. *Annali Di Igiene*.

[14] Decreto Ministero Sanità del 5 Luglio . Modificazioni alle istruzioni ministeriali 20 giugno 1896 relativamente all’altezza minima ed ai requisiti igienico-sanitari principali dei locali d’abitazione. 1975, https://www.indicenormativa.it/sites/default/files/Decreto%20Ministero%20della%20Sanita%2005-07-1975.pdf

[15] Gola M. (2017). Local health rules and building regulations: a survey on local hygiene and building regulations in Italian municiples. *Annali dell’Istituto Superiore di Sanità*.

[16] Legge 28 febbraio 1985, n. 47. Norme in materia di controllo dell’attività urbanistico-edilizia, sanzioni, recupero e sanatoria delle opere edilizie. (GU Serie Generale n.53 del 02-03-1985 - Suppl. Ordinario), https://www.gazzettaufficiale.it/atto/serie_generale/caricaDettaglioAtto/originario?atto.dataPubblicazioneGazzetta=1985-03-02&atto.codiceRedazionale=085U0047&elenco30giorni=false

[17] Mezzoiuso A. G. (2017). Ambienti confinati e salute: revisione sistematica della letteratura sui rischi legati all’utilizzo dei seminterrati a scopo abitativo. *Acta Biomedica*.

[18] Capasso L., Capolongo S., Faggioli A., Petronio M. G., Alessandro D. D.’ (2015). Do Italian housing regulations and policies protect poor people’s health?. *Annali Di Igiene*.

[19] Legge Regionale 21 novembre 2011, n. 21. Modifiche e integrazioni alla legge regionale n. 4 del 2009, alla legge regionale n. 19 del 2011, alla legge regionale n. 28 del 1998 e alla legge regionale n. 22 del 1984, https://www.regione.sardegna.it/j/v/80?s=181878&v=2&c=8824&t=1

[20] Corriere Della Sera. Cronache, https://www.corriere.it/cronache/13_novembre_18/maltempo-donna-muore-annegata-ad-oristano-894a6936-507f-11e3-b334-d2851a3631e3.shtml?refresh_ce-cp

[21] ISTAT, Popolazione residente—bilancio: sardegna, http://dati.istat.it/Index.aspx?QueryId=18975

[22] Arghittu A. (2018). Social deprivation indices and anti-influenza vaccine coverage in the Sardinian elderly population, Italy, with a focus on the Sassari municipality. *Journal Of Preventive Medicine and Hygiene*.

[23] Capasso L., Savino A. (2012). Analisi delle condizioni igienico-sanitarie di un campione di abitazioni del Comune di Chieti. *Annali Di Igiene*.

[24] European Commission (2004). *Reclaiming City Streets for People. Chaos or Quality of Life?*.

[25] Batur S., Teo T. (2014). Center and periphery. *Encyclopedia of Critical Psychology*.

[26] Kühn M. (2015). Peripheralization: theoretical concepts explaining socio-spatial inequalities. *European Planning Studies*.

[27] D’Alessandro D., Arletti S., Azara A. (2017). Strategies for disease prevention and health promotion in urban areas: the Erice 50 Charter. *Annali Di Igiene*.

[28] Capolongo S., Rebecchi A., Dettori M. (2018). Healthy design and urban planning strategies, actions, and policy to achieve salutogenic cities. *International Journal of Environmental Research and Public Health*.

[29] Decreto Del Presidente Della Repubblica 22 aprile 1994, n. 425 Regolamento recante disciplina dei procedimenti di autorizzazione all’abitabilita’, di collaudo statico e di iscrizione al catasto. (GU Serie Generale n.152 del 01-07-1994), https://www.gazzettaufficiale.it/eli/id/1994/07/01/094G0362/sg

[30] Carducci A., Fiore M., Azara A. (2019). Environment and health: risk perception and its determinants among Italian university students. *Science of The Total Environment*.

[31] World Health Organization (2019). *Violence and Injuries Prevention*.

[32] Dettori M., Arru B., Azara A. (2018). In the digital era, is community outrage a feasible proxy indicator of emotional epidemiology? The case of meningococcal disease in Sardinia, Italy. *International Journal of Environmental Research and Public Health*.

[33] Open Mind Research (2018). *La propensione al consumo di acqua del rubinetto in Italia*.

[34] Azara A., Muresu E., Dettori M., Ciappeddu P., Deidda A., Maida A. (2010). First results on the use of chloramines to reduce disinfection by products in drinking water. *Igiene E Sanità Pubblica*.

[35] Dettori M., Piana A., Castiglia P., Loria E., Azara A. (2016). Qualitative and quantitative aspects of drinking water supply in Sardinia, Italy. A descriptive analysis of the ordinances and public notices issued during the years 2010–2015. *Annali Di Igiene*.

[36] Azara A., Castiglia P., Piana A. (2018). Derogation from drinking water quality standards in Italy according to the European directive 98/83/EC and the legislative decree 31/2001—a look at the recent past. *Annali Di Igiene*.

[37] Dettori M., Azara A., Loria E. (2019). Population distrust of drinking water safety. Community outrage analysis, prediction and management. *International Journal of Environmental Research and Public Health*.

[38] Congiu T., Sotgiu G., Castiglia P. (2019). Built environment features and pedestrian accidents: an Italian retrospective study. *Sustainability*.

[39] Braubach M., Jacobs D. E., Ormandy D. (2011). *Environmental Burden of Disease Associated with Inadequate Housing*.

[40] ISTAT (2019). *Noi Italia. 100 Statistiche Per Capire Il Paese in Cui Viviamo*.

[41] D’Alessandro D., Buffoli M., Capasso L. (2015). Green areas and public health: improving wellbeing and physical activity in the urban context. *Epidemiologia E Prevenzione*.

[42] Rebecchi A., Buffoli M., Dettori M. (2019). Walkable environments and healthy urban moves: urban context features assessment framework experienced in milan. *Sustainability*.

[43] Azara A., Dettori M., Castiglia P. (2018). Indoor radon exposure in Italian schools. *International Journal of Environmental Research and Public Health*.

